# Selective Protection of an ARF1-GTP Signaling Axis by a Bacterial Scaffold Induces Bidirectional Trafficking Arrest

**DOI:** 10.1016/j.celrep.2014.01.040

**Published:** 2014-02-27

**Authors:** Andrey S. Selyunin, Lovett Evan Reddick, Bethany A. Weigele, Neal M. Alto

**Affiliations:** 1 Department of Microbiology, University of Texas Southwestern Medical Center, 5323 Harry Hines Boulevard, Dallas, TX 75390-8816, USA

## Abstract

Bidirectional vesicular transport between the endoplasmic reticulum (ER) and Golgi is mediated largely by ARF and Rab GTPases, which orchestrate vesicle fission and fusion, respectively. How their activities are coordinated in order to define the successive steps of the secretory pathway and preserve traffic directionality is not well understood in part due to the scarcity of molecular tools that simultaneously target ARF and Rab signaling. Here, we take advantage of the unique scaffolding properties of *E. coli* secreted protein G (EspG) to describe the critical role of ARF1/Rab1 spatiotemporal coordination in vesicular transport at the ER-Golgi intermediate compartment. Structural modeling and cellular studies show that EspG induces bidirectional traffic arrest by tethering vesicles through select ARF1-GTP/effector complexes and local inactivation of Rab1. The mechanistic insights presented here establish the effectiveness of a small bacterial catalytic scaffold for studying complex processes and reveal an alternative mechanism of immune regulation by an important human pathogen.

## INTRODUCTION

Membrane-associated pattern recognition receptors (PRRs), including Toll-like receptors, NOD-like receptors, RIGI receptors, and C-type lectins, recognize bacterial and viral pathogens and induce the expression of cytokines and chemokines that amplify the inflammatory response (Takeuchi and Akira, 2010). Although this system is highly effective in combating a diverse range of microbes, many bacterial pathogens have evolved strategies to overcome host defenses. In particular, Gram-negative bacteria use dedicated molecular machinery (e.g., type III secretion systems) that allows translocation of “effector proteins” into host cell cytoplasm (Galán and Collmer, 1999; Galán and Wolf-Watz, 2006). These molecules can covalently modify host signaling enzymes or directly mimic their regulatory components (Alto and Orth, 2012). Research over the past decade has focused primarily on identifying bacterial effector proteins that inhibit signal transduction cascades stimulated by the activation of PRRs (Baxt et al., 2013; Espinosa and Alfano, 2004). In contrast, only recently have researchers attempted to identify bacterial mechanisms that prevent cytokine and chemokine secretion by inhibiting vesicular transport through the general secretory pathway (GSP) (Burnaevskiy et al., 2013; Clements et al., 2011; Dong et al., 2012; Selyunin et al., 2011). Although arrest of protein transport would disable a wide variety of immune signaling pathways and therefore seems highly advantageous for pathogens, this strategy presents a challenge for bacteria that rely on host resources for survival (i.e., intracellular pathogens) and thus must be carefully orchestrated.

Cargo transport through the GSP follows a concerted route that includes the endoplasmic reticulum (ER), ER-Golgi intermediate compartment (ERGIC), and the Golgi apparatus. The packaging and delivery of transport vesicles between these compartments depends on microtubules and golgins, which control trafficking infrastructure and structural organization, and the function of ARF- and Rab-family GTPases, which play essential roles in regulating coat protein recruitment and budding, as well as tethering and fusion with target membranes, respectively (Donaldson and Jackson, 2011; Hutagalung and Novick, 2011). Like other members of the Ras superfamily, ARFs and Rabs cycle between active GTP-bound and inactive GDP-bound conformations. Exchange of GDP for GTP is mediated by guanine-nucleotide exchange factors (GEFs), whereas GTPase activating proteins (GAPs) stimulate hydrolysis of GTP to GDP (Cherfils and Zeghouf, 2013). In their active state, specific interactions of ARF and Rab GTPases with their downstream substrates define the molecular sequence of events that coordinate specific membrane trafficking events. Because the rapid turnover of GTPase signaling networks is essential for receptor localization and cytokine secretion, microbial regulation of host GTPases and their downstream interactions may be a powerful mechanism of immune evasion.

Recently, we discovered that the enterohemorrhagic *E. coli* (EHEC) type III bacterial effector protein EspG interacts directly with the GTP-active form of ARF1 and inhibits GAP-stimulated GTP hydrolysis (Selyunin et al., 2011). In addition, we found that EspG stimulated p21-activated kinase (PAK) through a nonoverlapping protein surface adjacent to the ARF1-binding site (Selyunin et al., 2011). In subsequent studies, Dong et al. (2012) showed that EspG functions as a Rab1-specific GAP through an endogenous TBC-like mechanism of action, despite having a unique structural fold. Interestingly, similar to what was observed for ARF1/PAK binding, EspG can simultaneously interact with ARF1 and Rab1. Together, these findings revealed a strong mechanistic connection underlying simultaneous recognition of multiple host proteins by EspG, and suggested that the scaffolding properties of a bacterial effector protein may allow selective control over signaling pathways at the Golgi apparatus. However, the significance of GTPase coupling through scaffolding properties has never been directly tested and the molecular mechanism of membrane trafficking regulation by EspG remains elusive. Considering the critical role of the GSP in innate immune function, we sought to delineate the biochemical significance behind simultaneous targeting of ARF1 and Rab1 signaling by EspG.

Here, we describe a model in which EspG arrests vesicular transport by stabilizing the ARF1-GTP tethering complex with simultaneous local inhibition of Rab1 signaling. By preventing GAP-mediated cycling of ARF1-GTP, EspG promotes the recruitment of ARF1-dependent tethering factors that restrict vesicle movement, whereas the Rab1-GAP activity of EspG further inhibits intracellular trafficking by preventing vesicle fusion. Importantly, we show that the scaffolding properties that allow simultaneous activity of EspG toward ARF1 and Rab1 GTPases are required for full potency during arrest of host intracellular trafficking.

## RESULTS

### EspG Disrupts Golgi through a Unique GTPase Regulatory Mechanism

The framework for understanding regulation of the GSP by ARF and Rab GTPases was previously established by studying cellular phenotypes resulting from their inactivation. In particular, our knowledge about ARF1 function in ER and Golgi trafficking was aided by the discovery of Brefeldin A (BFA), a fungal toxin that inhibits vesicle budding and transport by stabilizing an ARF1-GDP/GEF complex and preventing its transition into an active state (Klausner et al., 1992; Mossessova et al., 2003). Analogously, the roles of Rab1 signaling in membrane transport have been extensively studied using inhibition by GAPs and dominant-negative constructs (Haas et al., 2007). Importantly, inactivation of ARF1 or Rab1 signaling induces severe trafficking defects due to an arrest of ER export of proteins, manifested by accumulation of Golgi enzymes in the ER (Haas et al., 2007; Lippincott-Schwartz et al., 1989). However, the gross phenotypic changes associated with inactivation of an entire GTPase pathway by this method makes it difficult to understand the details of the numerous protein interactions that regulate Golgi function. In addition, because this approach targets individual pathways, it is not effective for elucidating the potential coordination of ARF1 and Rab1 signaling systems.

Detailed examination of a single bacterial effector molecule in the context of microbial infection has so far proven difficult, since type III secretion systems can deliver multiple effector proteins at any given time (Galán and Collmer, 1999). To overcome the problems associated with this complex system, we used micro-injection of purified recombinant protein, which allowed us to focus exclusively on the effects of EspG under physiologically relevant concentrations. When microinjected into cells, EspG induced fragmentation of the perinuclear Golgi ribbon and caused swelling of the ER-Golgi Intermediate Compartment (ERGIC), a complex system of tubulovesicular membrane clusters found near ER exit sites (Figure S1A). Considering that EspG arrests ARF1 GTP cycling (Selyunin et al., 2011) and displays Rab1-GAP activity (Dong et al., 2012), we set out to determine whether EspG functions by blocking ER export analogously to BFA and overexpression of eukaryotic Rab1-GAPs, or whether there exist important mechanistic differences between EspG and these independent GTPase inhibitors.

**Figure 1:**
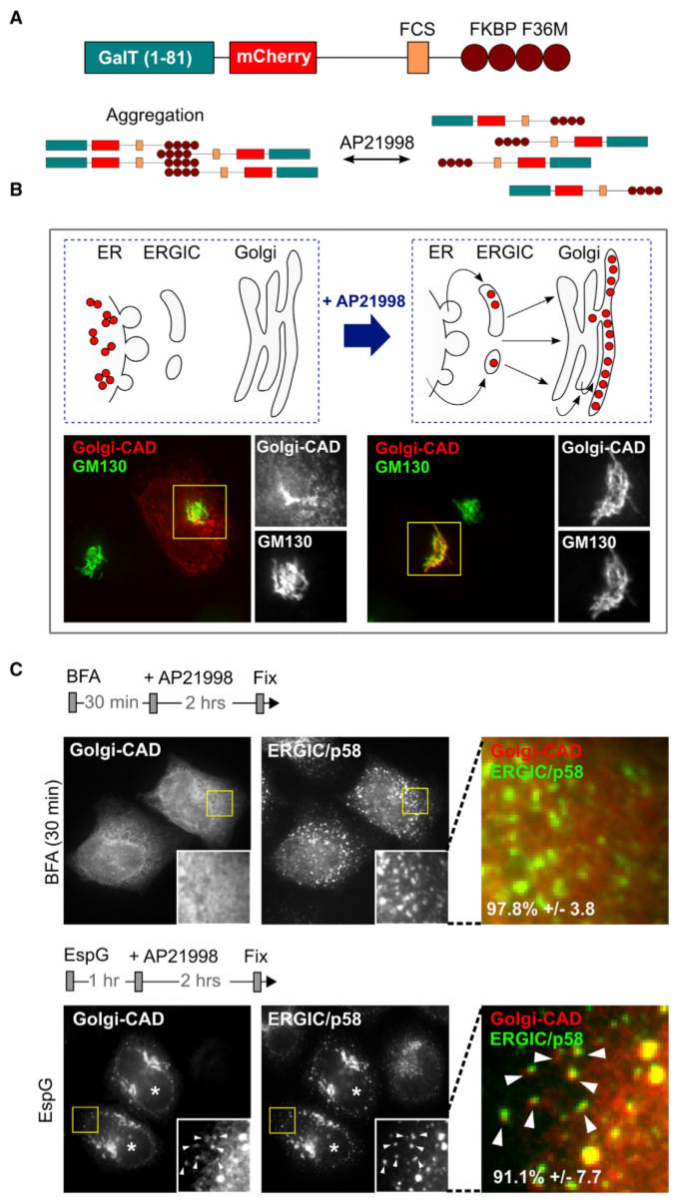
EspG Does Not Interfere with Protein Export from the ER (A) Schematic representation of the Golgi-CAD construct design. Aggregation of Golgi-CAD is mediated by FKBP F36M domains and can be reversed by addition of AP21998 molecule. (B) Development and confirmation of the inducible ER-to-Golgi trafficking assay to study protein transport through the early secretory pathway. The *trans*-Golgi-associated marker (Golgi-CAD) is trapped in the ER upon transfection (left), but is trafficked to the Golgi after the addition of the small molecule AP21998 (right). (C) Fluorescent micrographs show the final localization of Golgi-CAD 2 hr after addition of AP21998. Golgi-CAD escapes the ER but is arrested near p58-positive clusters (arrowheads) in cells microinjected with EspG (asterisks). No ER export occurs in cells treated with BFA. See also Figure S1.

To accurately address the effects of EspG on ER export, we developed a technique that tracks the movement of fluorescently labeled resident *trans-*Golgi enzyme β-1,4-galacto-syltransferase (GalT) through the secretory network, thus minimizing off-target effects, such as those associated with classic temperature-shift assays (Presley et al., 1997). The protein segment responsible for ER export and Golgi retention of GalT (residues 1–81) was translationally fused to the conditional aggregation domain (CAD) of a ligand-reversible crosslinking protein, FKBP F36M (Rivera et al., 2000), producing a marker we termed Golgi-CAD (Figure 1A). When transfected into cells, Golgi-CAD aggregated and was retained in the ER (Figure 1B, left). Addition of the small molecule AP21998 to the cell culture medium relieved aggregation and initiated normal trafficking of Golgi-CAD from the ER to the Golgi apparatus (Figure 1B, right).

To establish that the secretory pathway behaves normally under these conditions, Golgi-CAD was first allowed to accumulate in the ER, and then cells were treated with BFA for 30 min. AP21998 was added and Golgi-CAD localization was assessed after 2 hr. As expected, BFA potently inhibited ER export of Golgi CAD. Then, to determine whether EspG functions similarly, cells expressing Golgi-CAD were microinjected with EspG protein for 1 hr prior to AP21998 addition. Surprisingly, Golgi-CAD successfully exited the ER in EspG-injected cells but was arrested near vesicular clusters positive for p58, a marker associated with ERGIC (Figure 1C). These data indicate that EspG does not directly mimic the inhibitory mechanisms of BFA, as shown here, or overexpression of Rab1-GAP, as demonstrated previously (Haas et al., 2007). This notion was confirmed by additional studies on the medial Golgi enzyme N-acetylglucosaminyltransferase I (NAGT I) (Figure S1B).

### EspG Induces Trafficking Arrest Phenotypes Similar to Microtubule Disruption

**Figure 2:**
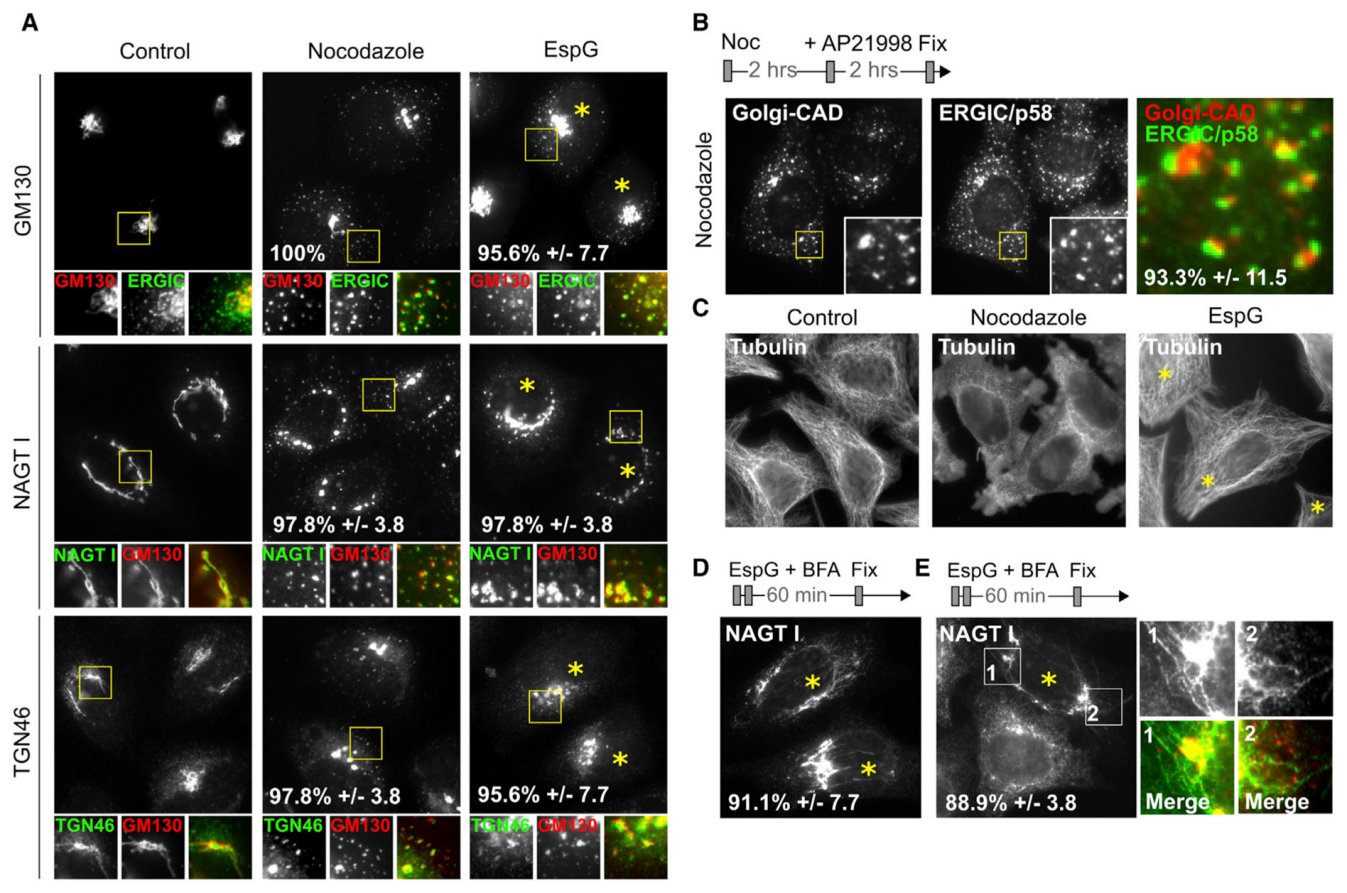
EspG Functionally Mimics Disruption of Microtubule-Dependent Processes (A) Fluorescent micrographs show Golgi morphology relative to positioning of *cis*- (GM130), medial- (NAGT I), and *trans*-Golgi (TGN46) markers in cells treated with nocodazole or microinjected with EspG. (B) Nocodazole prevents Golgi-CAD transport at p58-positive clusters during the inducible trafficking assay in a manner analogous to that observed for EspG. (C) EspG shows no apparent defect in microtubule network, despite the similar presentation of Golgi phenotypes. Microinjected cells are marked with an asterisk. (D) BFA-induced Golgi tubules persist in EspG-injected cells and fail to fuse with ER, suggesting inhibition of membrane transport along microtubules. Microinjected cells are marked with an asterisk. (E) Persistent BFA-induced Golgi tubules (green) emerge from and connect *cis*-Golgi (GM130, red) in cells transfected with EspG (asterisks). See also Figure S3.

Because EspG neither inhibited ER export nor induced accumulation of Golgi enzymes in the ER, we sought to define additional host factors that could complement its GTPase regulatory activities. In particular, EspG has been implicated in connection with host microtubules (Matsuzawa et al., 2004), which serve as tracks for vesicular transport (Cole et al., 1996). To examine the extent of this potential connection, we compared the distribution of cellular markers in cells treated with either EspG or nocodazole, a small-molecule inhibitor of microtubule polymerization. Nocodazole induced the redistribution of *cis*-(GM130), medial- (NAGT I), and *trans*-Golgi (TGN46) markers in a manner remarkably similar to that observed for EspG (Figure 2A). More importantly, during a Golgi-CAD trafficking assay, neither nocodazole nor EspG treatment blocked ER export, but instead arrested Golgi-CAD trafficking at the ERGIC, resulting in enlarged p58-positive clusters (Figure 2B). Surprisingly, however, no distinguishable defect in the microtubule network was found in cells microinjected with EspG, despite fragmented Golgi (Figure 2C). Thus, EspG appears to mimic the cellular effects of nocodazole by inhibiting membrane transport, albeit through a mechanism that is independent of the microtubule network.

To further investigate the extent of similarity between the EspG function and microtubule-dependent processes, we examined retrograde membrane transport from the Golgi to the ER. This step of retrograde transport can be readily studied in cells treated with BFA, which induces the formation of long Golgi membrane-tubules along microtubule tracks that rapidly fuse with ER membranes (Sciaky et al., 1997). Normally, Golgi tubulation and fusion are complete within 5–10 min of BFA addition. We took advantage of this phenotype to determine whether EspG could arrest retrograde trafficking of Golgi tubules. Cells were stimulated with BFA (to induce tubulation) and simultaneously microinjected with EspG. Remarkably, Golgi tubules that normally disappear within minutes of BFA treatment were instead completely arrested in the presence of EspG, and persisted even after 1 hr posttreatment (Figure 2D). Importantly, further cellular observations confirmed that these tubules extended from the *cis*-Golgi (Figure 2E). Because it is unlikely that the EspG family of proteins directly binds (Germane et al., 2008) or depolymerizes microtubules (shown in Figure 2C), we hypothesized that its mechanism is therefore dependent on the unusual properties manifested through simultaneous interactions with multiple host targets. Specifically, we focused on its binding to ARF1 and Rab1, considering their critical roles in ER-to-Golgi traffic and particularly their connection to microtubule-dependent cellular events (Horgan and McCaffrey, 2011).

### Structural Separation of ARF1 and Rab1 Regulatory Functions of EspG

**Figure 3:**
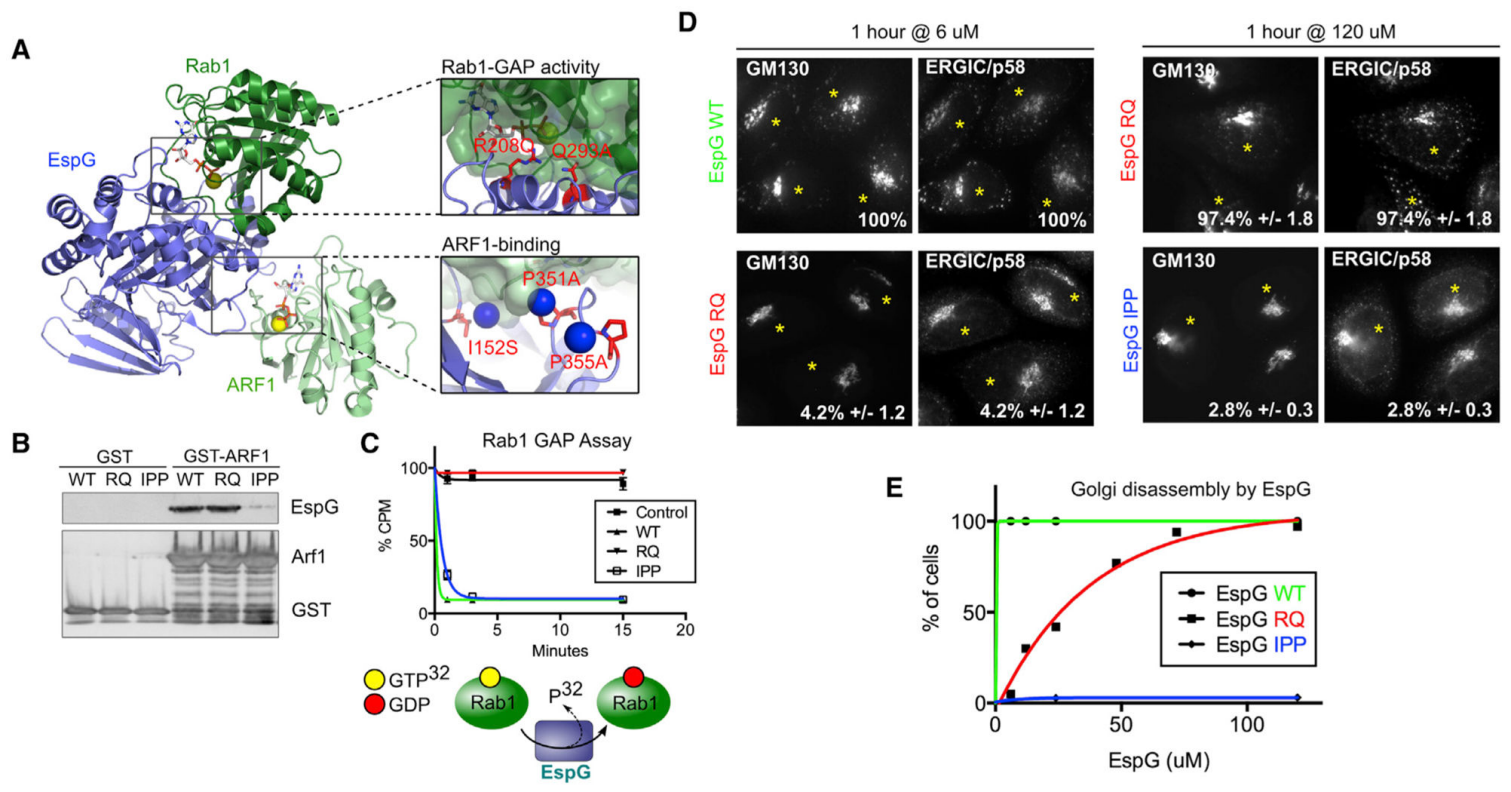
Separating the Roles of ARF1 and Rab1 in the EspG Mechanism (A) Identification of predicted ARF1- and Rab1-specific functional mutants based on structural data of EspG bound to ARF and Rab GTPases (3PCR, 4FMC, and 4FME). Mutations are shown in red, and blue spheres denote water molecules. (B) Pull-down experiments testing the ability of EspG mutants to bind ARF1. (C) Rab1-GAP assay testing the GAP activity of EspG constructs. (D) Golgi disassembly by EspG deficient for either ARF1 binding or Rab1-GAP activity as determined by Golgi ribbon fragmentation and enlarged p58 clusters. Representative micrographs are shown for the lowest and highest concentrations tested. Microinjected cells are marked with an asterisk. (E) Quantification of disrupted Golgi and p58 clusters phenotype. Needle concentrations are shown. See also Figures S2 and S3.

Although both structural and biochemical interaction data support the simultaneous binding of ARF1 and Rab1 to EspG (Dong et al., 2012), the relative contribution of each interaction to Golgi fragmentation and/or coordination with microtubule processes is unknown. To dissect their functional roles, we first generated EspG mutants that lacked either ARF1 binding or Rab1-GAP activity (Figure 3A). After a detailed analysis of available structural data and systematic mutagenesis, we identified an EspG mutant, I152S/P351A/P355A (EspG IPP), that was unable to bind ARF1 (Figure 3B) but retained potent GAP activity toward Rab1 (Figure 3C). Additionally, mutations in catalytic residues Arg-208 and Gln-293, which are required for EspG to function as a Rab1-GAP (Dong et al., 2012), had no defect in ARF1-GTP binding (Figures 3B and 3C).

Having created mutants specific for individual GTPases, we next compared their ability to produce changes in Golgi ribbon morphology and alter the appearance of p58-positive clusters to wild-type (WT) EspG protein. To accurately quantify this phenotype, we defined Golgi fragmentation as a relative spread of outermost Golgi marker appearance that exceeded 3 SDs from the spread measured in uninjected cells of the same experiment (Figure S2). Even at low cellular concentrations of protein, EspG WT induced Golgi disassembly and enlarged ERGIC in virtually every cell (100%; Figures 3D and 3E). In contrast, the ARF1-binding-deficient mutant, EspG IPP, did not disrupt Golgi architecture at any concentration tested (2.8% ± 0.3%), despite its ability to function as a Rab1 GAP (Figures 3D and S3). The cellular Rab1-GAP function of EspG may therefore require association with ARF GTPase in order to manifest the Golgi disruption phenotype. In agreement with this coordination of activities, the EspG RQ mutant, which was deficient for Rab1-GAP properties, induced Golgi fragmentation with significantly less potency than EspG WT (Figures 3D and 3E). However, increasing the concentration of microinjected EspG RQ resulted in an increase in the number of cells with fragmented Golgi (Figure 3E), suggesting that higher levels of ARF1 binding could compensate for the absence of its Rab1-GAP activity.

### EspG Disruption of Golgi Morphology Is PAK Independent

**Figure 4:**
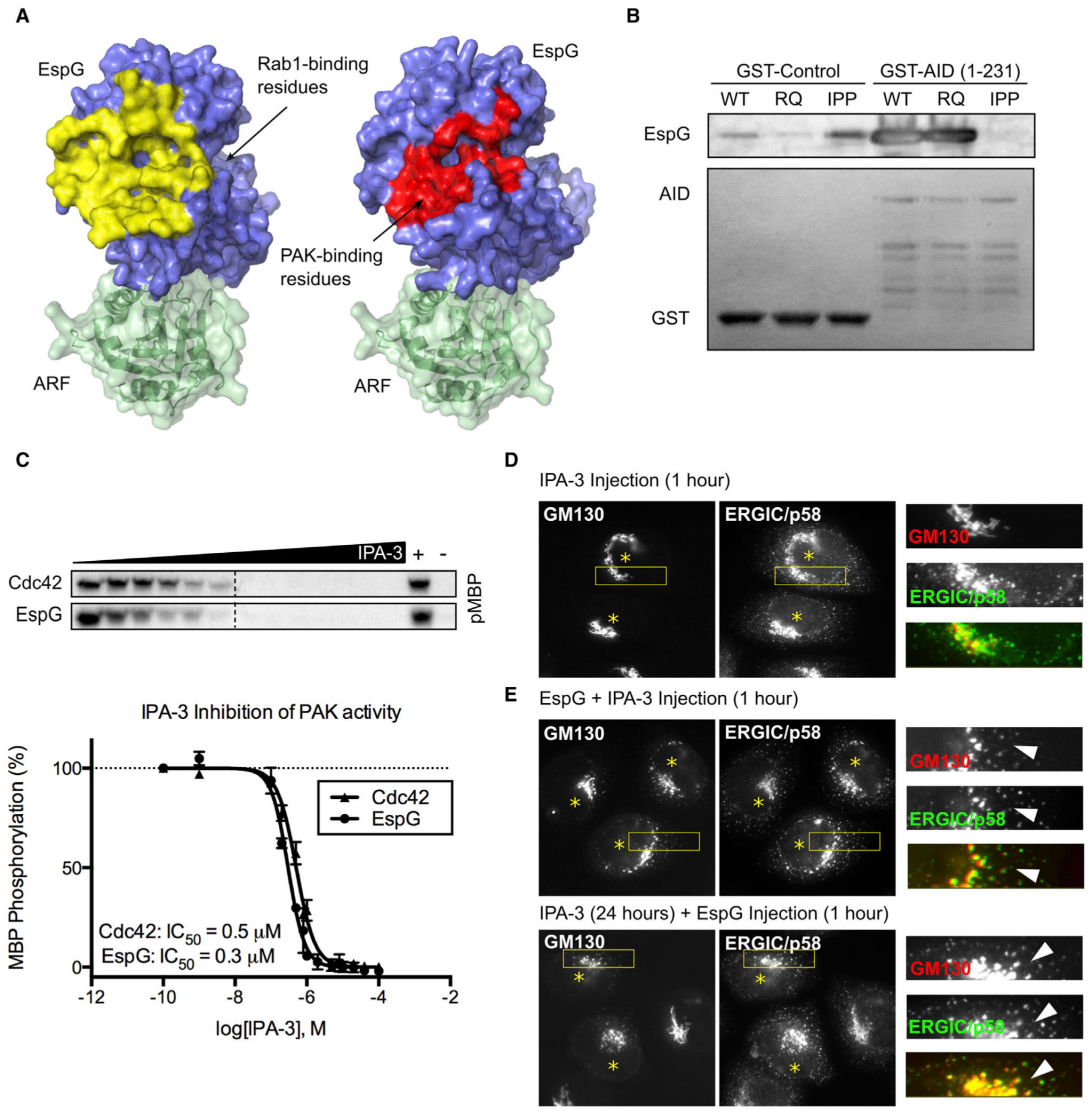
Fragmentation of the Golgi by EspG Is PAK Independent (A) PAK and Rab1 share the binding surface on EspG. Protein Data Bank structures 3PCS and 4FME were used for alignment. (B) Binding of EspG constructs to PAK AID. The ARF1-binding deficient mutant (IPP) does not interact with PAK. (C) Kinase assay showing the sensitivity of PAK activation to IPA-3. Radiography data from the assays were quantified and plotted to determine IC_50_ values. (D and E) Golgi fragmentation is induced by EspG in cells treated with IPA-3 to inhibit PAK. Microinjected cells are marked with an asterisk.

In addition to its ARF1-binding and Rab1-GAP properties, EspG directly stimulates PAK kinase activity (Selyunin et al., 2011). Interestingly, the binding sites for PAK and Rab1 share the same surface on EspG, suggesting that only one of the targets can bind at a time (Figure 4A). Therefore, regulatory scaffolding that is at the core of the EspG mechanism of Golgi disassembly could also involve ARF1/PAK complex. To address the role of PAK, we looked at the ability of EspG to induce Golgi fragmentation in the absence of PAK activity. When we tested EspG constructs for interaction with PAK autoinhibitory domain (AID; residues 1–241), we found that the WT and RQ mutants could effectively interact with PAK, whereas the ARF1-binding-deficient EspG IPP could not (Figure 4B). We were unable to isolate EspG mutants that were deficient for PAK activation, but not for ARF1 binding, likely due to linked structural features (Selyunin et al., 2011; A.S.S. and N.M.A., unpublished data). Thus, we could not address the roles of PAK versus Rab1 in EspG function using binding-specific mutants.

To overcome this difficulty, we used a PAK-specific small-molecule inhibitor (IPA-3) to globally inhibit PAK activity (Deacon et al., 2008). First, we confirmed that the EspG mechanism of PAK activation is sensitive to IPA-3 treatment. We found that EspG is susceptible to IPA-3 inhibition, even to a greater extent than observed for Cdc42 (IC_50_ = 0.3 μm M and 0.6 μM, respectively; Figure 4C). Next, cells were either microinjected with EspG/IPA-3 (50 μM) mix or incubated overnight in media containing 20 μM IPA-3 and then microinjected with EspG for 1 hr. In both cases, IPA-3 treatment had no effect on the ability of EspG to induce Golgi disassembly (Figures 4D and 4E). From these observations, we conclude that the ARF1-binding and Rab1-GAP activities of EspG, rather than PAK activation, that are responsible for disrupting the Golgi morphology. Taken together, our data indicate that cooperation between ARF1 and Rab1 signaling is required for full potency of EspG, and establish the EspG interaction with ARF1 as a key regulatory point in the EspG mechanism.

### EspG Functions Primarily through an ARF1 Signaling Axis

**Figure 5:**
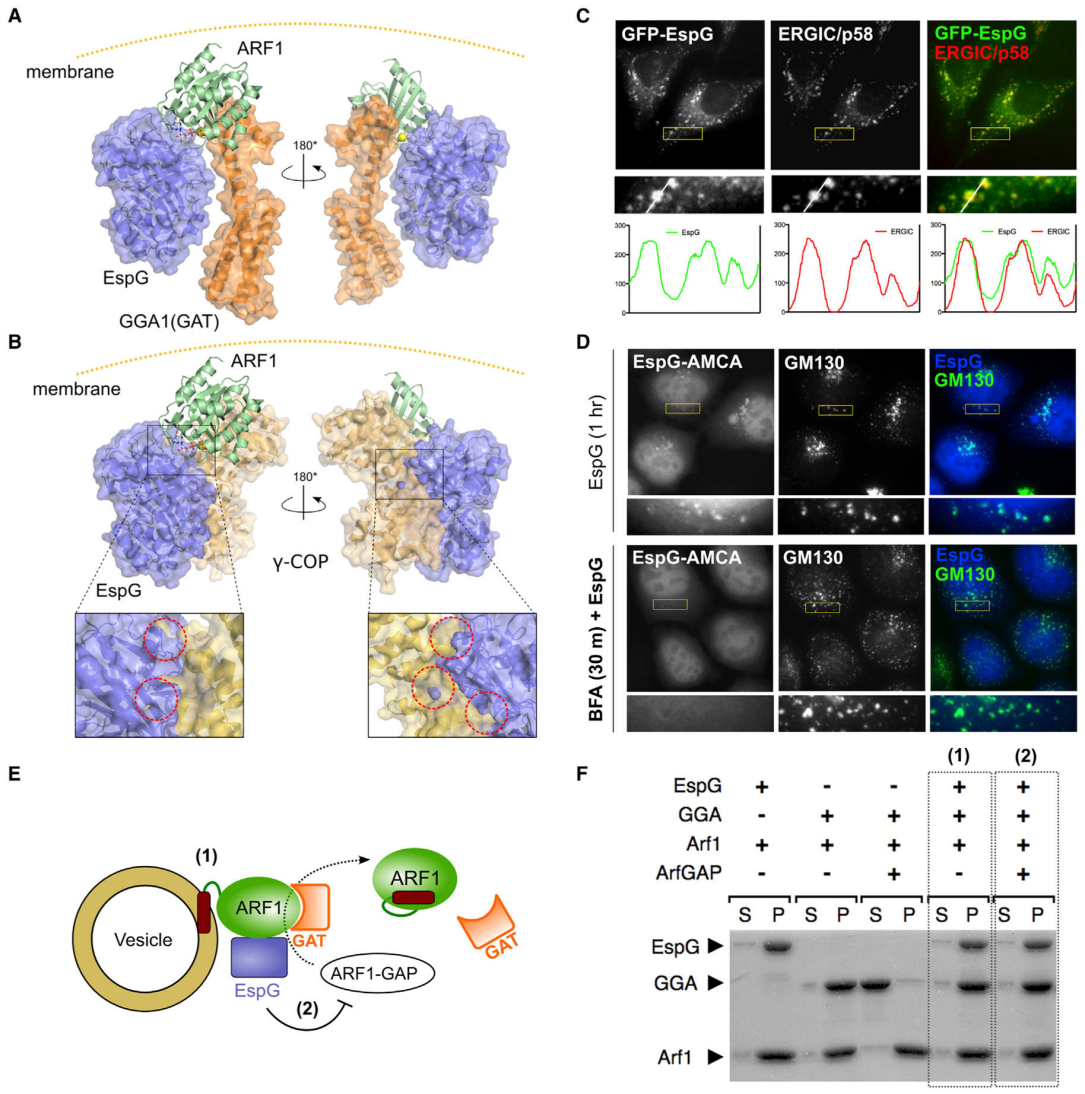
EspG Functions Selectively through Downstream Interactions of Active ARF1 (A) Structural model of EspG bound to the ARF1/GGA1(GAT) complex, based on EspG/ARF1 (1PCR) and ARF1/GGA1(GAT) (1J2J and1O3X) X-ray structures. (B) Structural model of EspG bound to the ARF1/γ-COP complex, based on EspG/ARF1 (1PCR) and EspG/γ, ζ-COP (3TJZ) structures. Insets show major steric clashes, highlighted with dotted circles. (C) Fluorescence micrographs show localization of GFP-EspG to p58-positive ERGIC membranes. (D) The membrane association of EspG is dependent on membrane-bound ARF1. Fluorescently labeled EspG is localized to fragmented endomembranes during microinjection, but is cytosolic when cells are pretreated with BFA. (E) Experimental setup to examine (1) membrane recruitment of downstream effectors by ARF1 and (2) the susceptibility of the ARF1/effector complex to GAP-mediated inactivation in the presence of EspG. (F) Liposome pull-down experiments for the setup described in (D). EspG allows recruitment of the ARF1 effector to membranes (1) and prevents its release due to ARF1 inactivation by ArfGAP (2). See also Figure S4.

In previous structural studies, we found that EspG occludes ARFGAP access to the GTP binding pocket of ARF1 by interacting at a distal site of the Switch I loop (Selyunin et al., 2011). Interestingly, this unusual binding architecture is distinct from known endogenous ARF1 substrate interactions, which primarily associate through the Switch II and β2/3 interswitch region (Nie et al., 2003). Thus, in addition to locking ARF in the GTP-active state, EspG may drive ARF1-GTP interactions with specific downstream substrates. Structural modeling revealed that EspG has no steric interference with the GAT domain of GGA1 adaptor protein when associated with ARF1-GTP (Figure 5A), and the GAT-Arf1-EspG complex is striking in its complementarity. In contrast, however, analysis of a recently solved structure of ARF1 in complex with γ-COP coat protein (Yu et al., 2012) revealed a significant clash between EspG and γ-COP (Figure 5B), indicating that EspG would interfere with COPI coat assembly at the Golgi. These observations suggest that the molecular architecture of EspG stabilizes the GTP-active state of ARF1 and, by protecting it from inactivation, effectively promotes select downstream signaling events. Therefore, we hypothesized that the pathogenic effects of EspG, and specifically those that regulate microtubule-dependent processes, are mediated by hyperstimulation of ARF1-GTP signaling.

To test this structural-based hypothesis, we first sought to determine whether EspG stabilizes an ARF1/substrate complex on host membranes. As shown in Figure 5C, EspG colocalized with p58, implicating Golgi membranes as the primary site of EspG function. Importantly, ARF1 was retained on p58-positive membranes in the presence of EspG, but not in cells treated with BFA (Figure S4A). These data are consistent with the biochemical differences between EspG and BFA: EspG locks ARF1 in the GTP active state, whereas BFA nucleates an inactive ARF1-GDP/GEF complex in the cytosol. Then, to determine whether EspG association with Golgi membranes requires ARF1-GTP Golgi localization, we assessed the sub-cellular distribution of fluorescently labeled EspG in either untreated cells or cells in which ARF1 was removed from membranes by BFA treatment. This experiment showed that EspG is cytosolic in the absence of membrane-bound ARF1, and thus requires ARF1-GTP for association with Golgi remnants (Figure 5D).

Next, we sought to determine directly whether membrane-bound GTP-ARF1 could recruit endogenous substrates to membrane bilayers when in complex with EspG. Initially, we reconstituted this system on Golgi-mimetic liposomes using purified recombinant proteins (Figure 5E). In control experiments, the soluble GAT domain of GGA1, a prototypic ARF1 substrate that binds with high affinity, was selectively pulled down by liposomes carrying GTP-loaded ARF1 (Figure 5F, lanes 3–6). In agreement with our structural predictions, GGA1(GAT) was successfully recruited into the EspG/ARF1 complex in an ARF1-GTP-dependent manner (Figure 5F, lanes 7 and 8). Importantly, EspG blocked the GAP-stimulated release of GGA1(GAT) from liposomes, indicating that EspG effectively induces a constitutively active form of ARF1 on membranes (Figure 5F, lanes 9 and 10). In cell-based studies, we also found that GGA1(GAT) was recruited to fragmented Golgi membranes in cells microinjected with EspG, but not in cells where Golgi fragmentation was induced by BFA (Figure S4B). As predicted by our structural model, EspG interfered with β-COP recruitment to membranes in a subset of cells (Figure S4C), but not in all cells (Selyunin et al., 2011). It is possible that some coatomer assembly and vesicle formation still occurred due to the considerable excess of ARF1 molecules relative to EspG, as well as ARF-independent COP recruitment to membranes (Deng et al., 2009; Ktistakis et al., 1996). Taken together, these data strongly support a mechanism whereby EspG selectively protects specific downstream interactions of membrane-bound ARF1-GTP, which combined with inactivation of Rab1 leads to Golgi disassembly and inhibition of the GSP.

### Identification of Downstream ARF1 Effectors

So far, our data indicate that EspG initiates an ARF1 GTPase signaling cascade that is either directly or functionally linked to microtubule-dependent membrane transport. Because neither EspG nor ARF1 binds microtubules, the molecular mechanism of EspG must therefore involve a host cellular component that is (1) a specific effector for ARF1, (2) required for Golgi structural maintenance, and (3) capable of inducing Golgi fragmentation without affecting the microtubule network. Through extensive analysis of published observations, we found two proteins that met these criteria: GMAP-210 and Golgin-160. GMAP-210 is a tubulin-binding protein that links small vesicles to organelle membranes via a curvature-sensing N-terminal ArfGAP1 Lipid Packing Sensor (ALPS) and a C-terminal GRIP-Related ARF Binding (GRAB) domain that interacts with ARF1-GTP (Drin et al., 2008; Ríos et al., 2004). Golgin-160, on the other hand, is found primarily on the *cis*-Golgi and is implicated in membrane transport by connecting ARF1-GTP vesicles to dynein motors (Yadav et al., 2012). Although the microtubule network remained intact, depletion of either GMAP-210 or Golgin-160 disrupted the Golgi morphology and resulted in a dispersed ministack appearance of the Golgi (as visualized by transmission electron microscopy), similar to the Golgi phenotype observed following nocodazole treatment (Pernet-Gallay et al., 2002; Ríos et al., 2004; Yadav et al., 2009).

**Figure 6:**
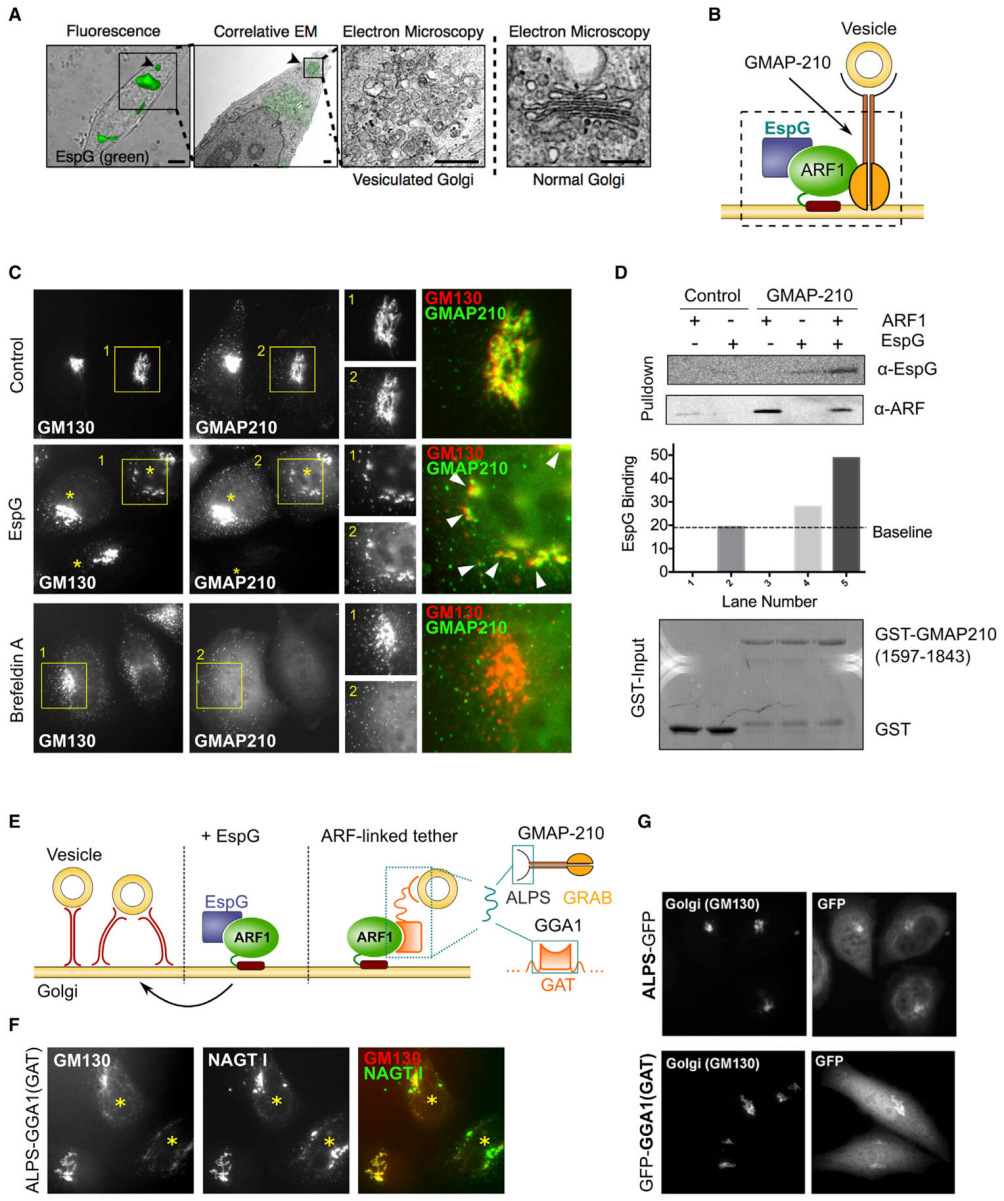
Disruption of the Golgi Architecture through ARF1-Dependent Membrane Tethering (A) CLEM shows the vesicular ultrastructure of the Golgi after microinjection with EspG, which remains constrained to areas positive for EspG signal. (B) Identification of golgin GMAP-210 as an ARF1-dependent membrane tether involved in the EspG mechanism of Golgi disassembly by linking vesicles to membranes and preventing their transport. (C) Fluorescent micrographs showing the distribution of GMAP-210 relative to the Golgi (GM130). GMAP-210 is retained on fragmented membranes following disassembly of the Golgi by EspG (arrowheads), in contrast to when ARF1 is removed from the membranes by BFA treatment. (D) Pull-down experiment showing the binding of EspG to GMAP-210/ARF1 complex. (E) Cartoon schematic illustrating the design of an ARF1-dependent, membrane-tethering chimera using a vesicle-binding motif linked to an ARF1-GTP-binding domain of GGA1. Our model of EspG function predicts vesicle-to-membrane linkage that is driven by the presence of GTP-ARF1 on membranes. (F) Overexpression of the ARF1-dependent, membrane-tethering chimera is sufficient to disrupt the Golgi architecture. Transfected cells are marked with an asterisk. (G) Control experiments show no impact on Golgi organization due to overexpression of either a vesicle-binding motif or an ARF1-GTP-binding domain alone in the absence of a direct tether. See also Figure S5.

Because cellular disruption of GMAP-210 and Golgin-160 resulted in a well-defined multivesicular Golgi phenotype, we used correlative light electron microscopy (CLEM) to determine whether EspG would induce a similar Golgi morphology. The cellular membrane fragments that correlated with the EGFP-EspG fluorescent signal were organized in clusters of small vesicles (Figure 6A). These vesicles did not stochastically spread throughout the cell, suggesting that the membranes may be crosslinked through an EspG/ARF1-GTP-mediated protein complex. Based on our observations that ARF1-binding by EspG is sufficient to induce Golgi fragmentation, and EspG stabilizes ARF1-GTP on membranes, we hypothesized that EspG most likely blocks vesicular traffic through an ARF1-dependent tether (i.e., GMAP-210 or Golgin-160; Figure 6B). Importantly, this mechanism would explain the phenotypic similarity between EspG and nocodazole treatment, as both restrict vesicular movement beyond their budding site (Cole et al., 1996).

### EspG Functions through GMAP-210 Signaling from the ARF1 Complex

We now propose a model in which EspG promotes membrane tethering by stabilizing ARF1-GTP on Golgi membranes, which effectively increases the presence of tethering factors such as GMAP-210 and inhibits vesicle fusion through Rab1-GAP function. This model predicts that excessive expression of GMAP-210 should induce a Golgi phenotype analogous to EspG. To test this assumption, we examined the effects of excess GMAP-210 on Golgi structure and function. Transient overexpression of the tethering protein induced severe Golgi fragmentation and enlarged ERGIC (Figure S5A), as well as accumulation of Golgi enzymes near p58-positive clusters (Figure S5B). Similar to what was observed for EspG, Golgi disassembly by GMAP-210 overexpression was not due to depolymerization of microtubules, which appeared intact (Figure S5C). We then wanted to confirm that inhibition of protein transport due to excessive GMAP-210 tethering occurred in a manner consistent with what we observed during EspG microinjection. Indeed, we found that Golgi-CAD was similarly trapped near p58-positive clusters during an anterograde trafficking assay (Figure S5D), and that overexpression of GMAP-210 also protected NAGFP from BFA-induced redistribution into the ER (Figure S5E). Finally, EM analysis of Golgi remnants during over-expression of GMAP-210 showed a striking similarity to those observed with EspG overexpression, displaying an extensive accumulation of vesicles (Drin et al., 2008; Figure 6A).

At the core of our model, EspG protects membrane-bound ARF1-GTP from inactivation by ARF GAP, increasing the selective accumulation of downstream ARF1-GTP substrates (i.e., GMAP-210) that promote vesicle tethering and inhibit membrane fusion. This suggests that inhibition of membrane trafficking associated with EspG is due to extensive ARF1-dependent tethering events between membranes and vesicles (Figure 6E). To more directly confirm that ARF1-dependent vesicle tethering is sufficient to induce Golgi disassembly, we reengineered GMAP210 and GGA1-GAT to model the proposed EspG virulence system with a minimal set of interactions. Specifically, we created a synthetic chimera protein (ALPS-GAT) consisting of a GMAP-210 ALPS motif to bind small vesicles, and a soluble GGA1-GAT domain, which associates with membranes only when bound to active ARF1-GTP (Figure 6E). If our model were correct, then the minimal ALPS-GAT chimera would functionally mimic two aspects of the proposed EspG/ARF1/GMAP210 complex: (1) it would tether vesicles to Golgi membranes in an ARF1-dependent manner, and (2) it would selectively promote tethering while restricting other ARF-GTP signaling events, such as COPI vesicle fission. In agreement with our model, cellular expression of ALPS-GAT effectively disrupted Golgi organization and function in a manner similar to that observed for EspG (Figure 6F), but distinct from the phenotype we have reported for BFA. Importantly, expression of either the ALPS motif or GAT domains alone had no effect on Golgi morphology, confirming that disruption was directly dependent on tethering of small vesicles to membrane-bound ARF1-GTP (Figure 6G).

Because in vitro assays to examine direct interactions between GMAP-210 and ARF1 have proved difficult (Gillingham et al., 2004), we used structural modeling to assess whether EspG could bind to the ARF1/GMAP-210 complex on the membrane. Using the structure of a related GRIP domain of golgin-245 bound to Arl1 (Panic et al., 2003), we modeled the ARF1/ GMAP-210 interaction with EspG, and did not detect any apparent steric clashes (Figure S5F). To test our hypothesis more directly, we next looked at the presence of the ARF1/ GMAP-210 complex on fragmented Golgi remnants after EspG microinjection. The C-terminal portion of GMAP-210 that contains the ARF-binding motif (residues 1597–1843) localizes primarily to the Golgi in an ARF1-dependent manner and does not cause any apparent defects in Golgi morphology (Figure 6C). As expected, BFA treatment abolished the association of GMAP-210 (residues 1597–1843) with the membranes, in contrast to EspG, whose microinjection retained membrane-bound GMAP-210 despite fragmented Golgi (Figure 6C). Finally, we also found that EspG associated with the ARF1/GMAP-210 complex in in vitro glutathione S-transferase (GST)-pull-down experiments (Figure 6D). Together, these observations suggest that the ARF1/GMAP-210 complex has a major role in the EspG mechanism.

**Figure 7:**
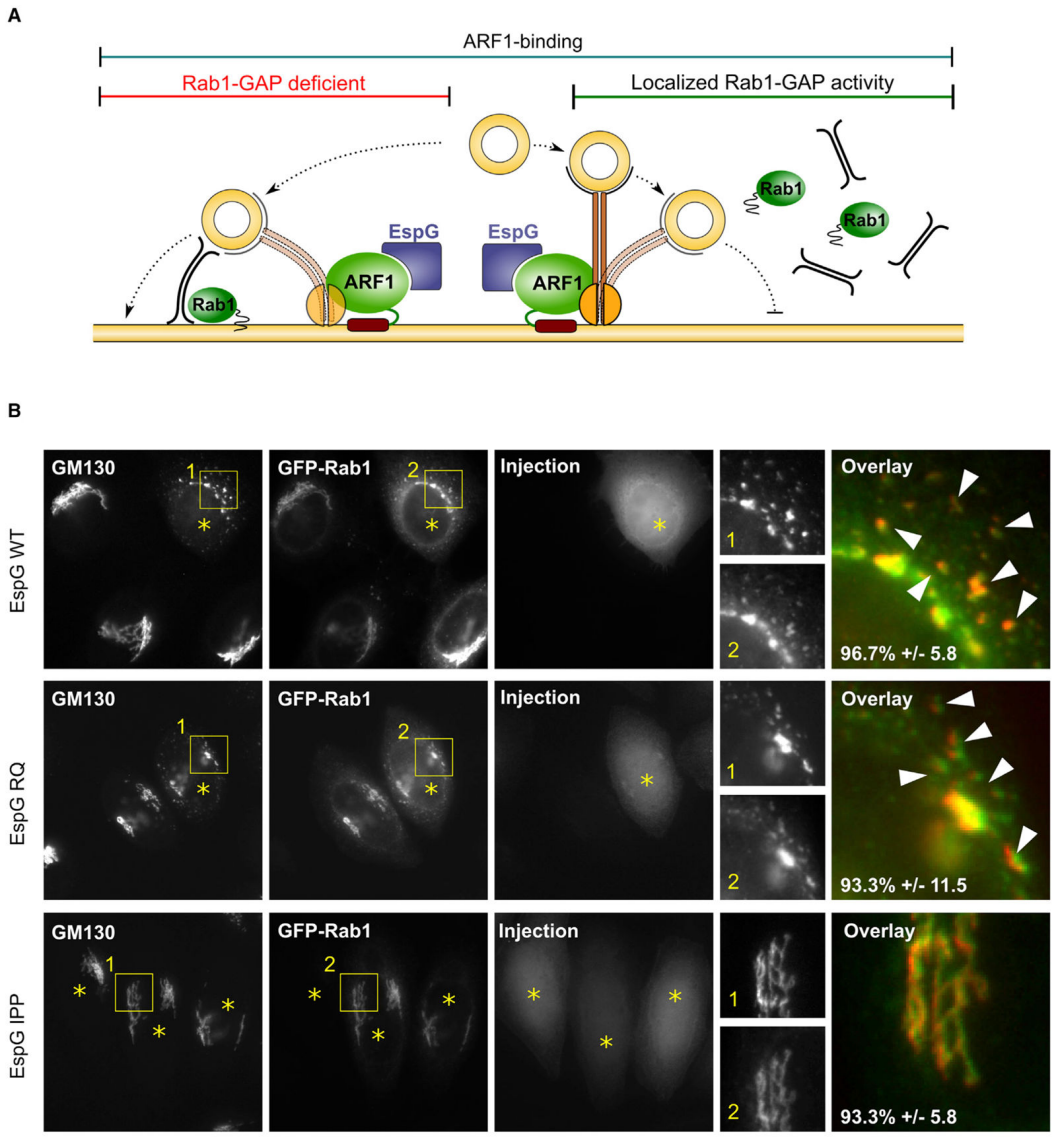
EspG Inhibits Membrane Trafficking through Membrane Capture and Local Inactivation of Rab1 (A) Cartoon model of the EspG mechanism that relies on ARF1-dependent tethering and exclusion of Rab1-dependent vesicle fusion machinery. (B) Fluorescent micrographs showing the localization of Rab1 relative to Golgi membranes in cells microinjected with EspG.

Thus, we predict that EspG binding to ARF1 drives excessive membrane accumulation of specific ARF1 substrates, such as GMAP-210, which consequently would lead to increased tethering of vesicles or allow regulation of additional microtubule-dependent processes (Figure 7A). Rab1-GAP activity prevents vesicle fusion via Rab1-dependent machinery (Figure 7A, left), which otherwise would require significantly higher levels of EspG-induced tethering in order to outcompete and physically restrict membrane fusion. Indeed, this phenomenon is reflected in the attenuated induction of Golgi morphology by the EspG RQ mutant (see Figure 4E). Moreover, ARF1 binding spatially restricts the Rab1-GAP properties of EspG to a specific site, which could be connected to the observed phenotypic differences between EspG and Rab1-GAP transfection that led to a global loss of Rab1 signaling. As expected, Rab1 did not fully dissociate from fragmented Golgi in cells microinjected with EspG (Figure 7B). By preventing vesicle fusion and capturing vesicles in close proximity to membranes, EspG effectively inhibits vesicle transport at ERGIC and leads to Golgi disassembly.

## DISCUSSION

In its active state, ARF1 recruits a wide variety of effectors to membranes, including COPI coat proteins, lipid-modifying enzymes, and scaffolds for cytoskeletal anchoring (Donaldson and Jackson, 2011). This makes it a prime target for a bacterial protein that has evolved to regulate intracellular trafficking. Although restricting GAP access allows EspG to control the activity state of ARF1, maintaining the GTPase in a constitutively active confirmation through allosteric binding alone would be inefficient when compared with molecules mimicking upstream regulatory proteins, such as GEFs. Instead, the binding nature of EspG suggests that it additionally acts as a selection mechanism that allows interaction of ARF1 with specific, but not all, downstream substrates. Indeed, some endogenous ArfGEFs have been shown to favor specific downstream signaling by interacting with and preferentially recruiting a set of effectors to ARF1 (Deng et al., 2009). Downstream substrate selectivity combined with protection of ARF1 from GAP inactivation would effectively allow EspG to inhibit some signaling pathways while overstimulating others, thus expanding its function beyond that of a simple activity modulator. Our proof-of-concept experiments with Golgi mimetic liposomes support this idea by showing that ARF1 can recruit its effectors to membranes when bound by EspG, and that this complex remains resistant to GAP treatment.

Interestingly, EspG displays a clear distinction from BFA-or Rab1-GAP-associated trafficking phenotypes, as it neither inhibits ER export nor leads to accumulation of Golgi enzymes in the ER. This suggests that regulatory coupling of host signaling enzymes through scaffolding architecture, rather than individual regulatory properties, drives the mechanism of EspG. Targeting multiple host regulatory pathways through a single effector molecule would allow bacteria to more efficiently modulate complex host signaling networks and finely tune a specific environment for their lifestyle. Indeed, an EspG homolog from the intracellular pathogen *S. flexneri*, VirA, shares potent GAP activity toward Rab1 and induces Golgi disassembly (Dong et al., 2012). In contrast to EspG, however, VirA does not interact with ARF1, and instead may coordinate the inactivation of Rab1 with a different signaling pathway, further supporting specific evolutionary adaptations of effector proteins. Notably, Golgi disruption by *S. flexneri* can also be independently induced by the recently characterized IpaJ effector, which demyristoylates and inactivates ARF1 (Burnaevskiy et al., 2013). It is therefore possible that IpaJ and VirA activities complement each other to precisely control a range of trafficking events under different conditions, further underlining the functional need to selectively coordinate the regulation of individual pathways.

Elucidating the exact functional role of an individual bacterial effector molecule in an infection is difficult due to the large number of proteins that are secreted by invading bacteria, the variable number of bacteria that infect individual cells, and the poorly understood order of secretion of effectors, some of which may share host targets or mask activity. These factors introduce wide variance and make it hard to connect a distinct phenotype to any single molecule. By understanding the molecular mechanism of an effector through focused biochemical studies, such as the one presented here, we can uncover its potential function within a host cell and obtain direct insight into its role during an infection. Specifically, EspG and VirA have been linked to microtubule cytoskeleton phenotypes in cells infected by EHEC or *S. flexneri*, respectively (Matsuzawa et al., 2004; Yoshida et al., 2002). Here, we show that a distinct trafficking phenotype appears closely related to that observed during microtubule disruption by nocodazole, and that this connection could be linked to the regulation of ARF and Rab GTPases. In addition, EHEC infection has been shown to inhibit cytokine secretion and decrease transepithelial resistance (Dong et al., 2012; Philpott et al., 1998). We present evidence that EspG potently inhibits GSP by arresting membrane transport at ERGIC, which in turn inhibits cytokine secretion and delivery of adherence proteins needed to maintain the tight junctions at the cell surface in the intestinal lumen.

Simultaneous regulation of select trafficking pathways, such as antigen presentation by major histocompatibility complex class I molecules, immunoglobulin M secretion, autophagosome formation, and cytokine secretion, may afford bacterial pathogens the ability to avoid immune recognition specific to their lifestyle. Indeed, EspG has been found to inhibit interleukin-8 secretion from HeLa cells during infection (Dong et al., 2012). At the same time, the wide variety of bacteria with distinct lifestyles provide researchers with effective tools to better understand the signaling interplay within complex regulatory systems by studying the mechanisms of secreted scaffolding effector proteins. Taken together, our data expose signaling-pathway coupling via small molecular scaffolds as a regulatory hub of intracellular trafficking, and describe an additional level of structural specificity that may have been previously overlooked.

## EXPERIMENTAL PROCEDURES

### Plasmids

The *espG* gene from EHEC O157:H7 was PCR cloned in frame into pEGFP-C2 (Clontech). For bacterial expression, 38 and 41 aa N-terminal deletions (residues 39–398 and 42–398) of EspG were PCR subcloned into pGEX-4T1 (GST-tag; Amersham) and pProEX-HTb (6×His tag; Novagen) vectors. EspG mutants were generated with QuikChange site-directed mutagenesis (Stratagene). Full-length ARF1 or the GTPase domain of ARF1 (ARF1Δ17) were PCR subcloned into pcDNA3.1-mCherry or pGEX-4T1 and pProEX-HTb vectors, respectively. The GTPase domain of Rab1 (residues 1–177) or full-length ARF1 was subcloned from Rab1a cDNA (DNASU, Arizona State University) into pGEX-4T3 or pEGFP-C1 vectors (Clontech). The GMAP-210 plasmid was a kind gift from Bruno Antonny and Guillaume Drin (Institut de Pharmacologie Moléculaire et Cellulaire). All constructs were verified by DNA sequencing.

### Protein Purification for In Vitro Assays and Microinjection

Recombinant proteins were produced in BL21-DE3 *E. coli* strains. Protein expression was induced with 0.4 mM isopropyl β-D-1-thiogalactopyranoside for 16 hr at 18°C. Bacterial pellets were lysed in either His buffer (100 mM HEPES [pH 7.5], 300 mM NaCl) or GST buffer (TBS; 50 mM Tris [pH 7.5], 150 mM NaCl, 2 mM dithiothreitol) supplemented with protease inhibitors. Proteins were purified with nickel agarose (QIAGEN) or glutathione Sepharose (Amersham Biosciences). For microinjection, protein stocks were diluted with TBS to the indicated concentrations.

### In Vitro GST Pull-Downs

Recombinant GST-tagged proteins (10 μg) were immobilized to glutathione Sepharose and incubated with 15 μg of 6×Histagged proteins for 1 hr at 4°C. Samples were washed three times in TBS supplemented with 0.5% Triton X-100. Proteins were eluted from beads with Laemmli sample buffer, separated by SDS-PAGE, and analyzed by western blot. For nucleotide loading, ARF1Δ17 was incubated in nucleotide loading buffer (40 mM HEPES, 150 mM NaCl, 2 mM EDTA, 10% glycerol) with 10 μM of either GDP or GTP for 30 min at 37°C, and then MgCl_2_ was added to 10 mM and the reaction was transferred to ice after 15 min at 25°C.

### Cell Microinjection, Transfections, and Immunofluorescence Microscopy

HeLa cells were microinjected with EspG proteins using a semiautomatic InjectMan NI2 (Eppendorf) with a needle concentration of 25 μM unless stated otherwise. Transfections were performed using XtremeGene 9 Transfection Reagent (Roche) for 16–18 hr. Expression of NAGT I in NAGFP cells was stimulated by the addition of 5 μM sodium butyrate (Sigma-Aldrich) to the media. AMCA-EspG was produced using an NHS-AMCA labeling kit (Pierce). BFA and nocodazole treatments were performed at 5 μg ml^−1^ for 30 min and 30 μM for 2 hr, respectively. Cellular markers were detected using following antibodies: GM130 (BD Transduction Labs), ERGIC-53/p58 (Sigma-Aldrich), TGN46 (Abcam), β-COP (EAGE, Joachim Seeman, UTSW), and α-tubulin (Sigma-Aldrich).

### Ligand-Inducible ER-to-Golgi Trafficking Assay

An inducible trafficking assay was developed by adapting the CAD of FKBP F36M (Rivera et al., 2000) to control the secretory transport of a fluorescently tagged β-1,4-galactosyltransferase signal sequence. See the Supplemental Experimental Procedures for details.

### Liposome Pull-Downs and Rab1 GAP Assay

Golgi mimetic liposomes were generated using lipid ratios reported by Bremser et al. (1999). GTP loading of ARF1, liposome pull-down assays, and GAP assays were conducted as previously described (Selyunin et al., 2011). Briefly, ARF1-GTP-containing liposomes were incubated with GGA1(GAT) in the absence or presence of EspG and ArfGAP, as shown in Figure 5, and binding was assessed by SDS-PAGE after sedimentation. In the Rab1-GAP assay, 5 μM GST-Rab1 was incubated with 1 μM EspG (WT, RQ, and IPP) for 15 min at 30°C. See the Supplemental Experimental Procedures for additional details.

### PAK Kinase Assay and IPA-3 Sensitivity

Kinase assays were performed as previously described (Selyunin et al., 2011). In brief, GFP-PAK2 was purified from HEL293 cell lysates and preincubated with myelin basic protein and the indicated amounts of IPA-3 (Sigma-Aldrich) or DMSO in kinase buffer for 20 min at 4°C. Cdc42-GTPγS or EspG (2 μM) was then added and the reaction was preequilibrated for 10 min at 30°C. Reactions were started by the addition of ATP with [^32^P]ATP and incubated for 15 min. Finally, reactions were analyzed by SDS-PAGE and autoradiography, and quantified using a scintillation counter.

### CLEM

Microinjection and CLEM were performed in a manner similar to that described in Reddick and Alto (2012). Briefly, HeLa cells were cultured on gridded glass coverslips (MatTek). EspG was fluorescently labeled using fluorescein isothiocyanate labeling (Pierce). Cells were fixed with 3% formaldehyde in PBS 30 min postinjection and a confocal Z stack was acquired on microinjected cells using a Zeiss LSM5 Pascal confocal microscope. CLEM was performed exactly as described in Reddick and Alto (2012).

## Supplementary Material

01
