# Supplementary material for: Selective Protection of an ARF1-GTP Signaling Axis by a Bacterial Scaffold Induces Bidirectional Trafficking Arrest
Source: Cell Rep. Author manuscript; Available in PMC 2014 May 12. (PMC4017587; doi:10.1016/j.celrep.2014.01.040)
Supplement: 01 [file NIHMS562520-supplement-01.pdf]

Selective protection of an ARF1-GTP signaling axis by a bacterial scaffold induces bidirectional trafficking arrest.

Andrey S. Selyunin, L. Evan Reddick, Bethany A. Weigele, and Neal M. Alto

Supplemental Figures S1-S6: p.2-6

Author Contributions: p. 7

Experimental Procedures: p. 8-9

Figure S1

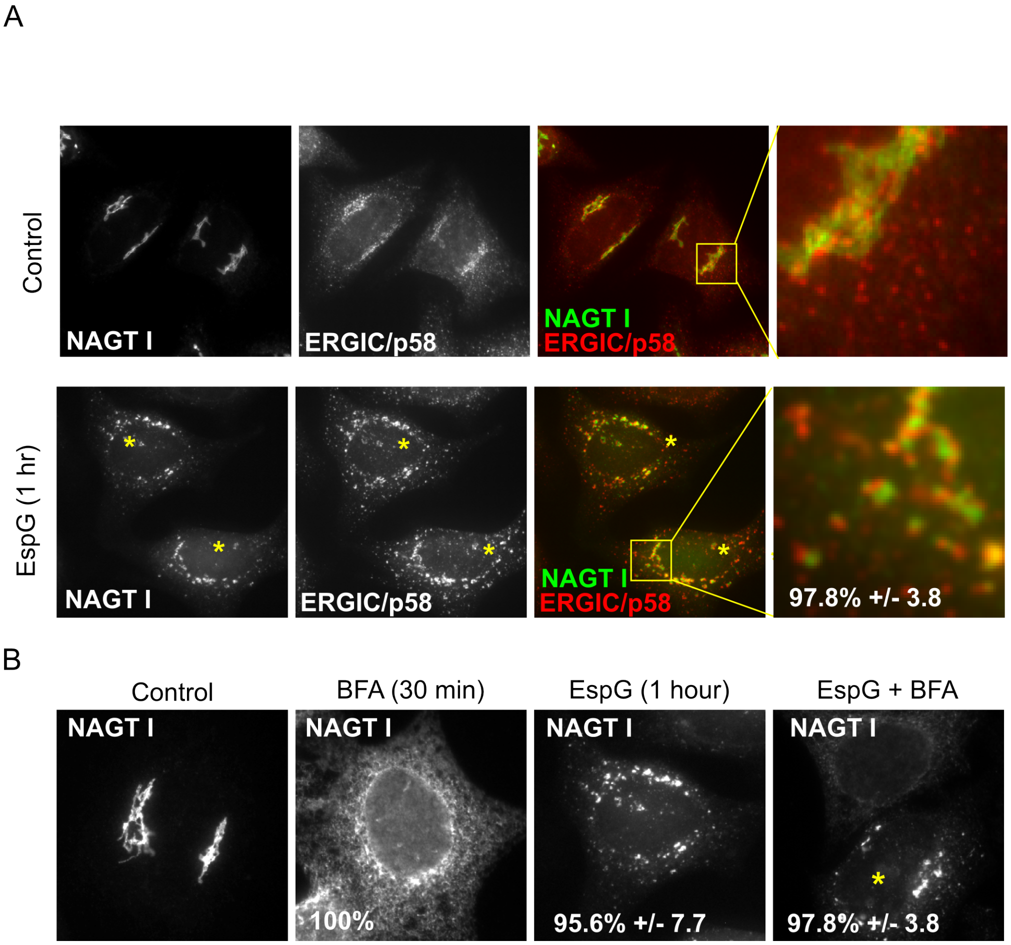

Figure S1. EspG fragments Golgi into perinuclear structures near p58 clusters, related to Figure 1.

(A) EspG induces fragmentation of Golgi ribbon into clusters within close proximity to ERGIC associated membranes. Injected cells are marked with asterisks.

(B) BFA treatment leads to rapid accumulation of Golgi enzymes in ER. Despite also targeting ARF1 cycling, EspG arrests Golgi enzymes near p58 clusters instead. Moreover, microinjection of EspG (asterisk) protects Golgi enzymes from redistribution into ER even after consecutive treatment with BFA.

Figure S2

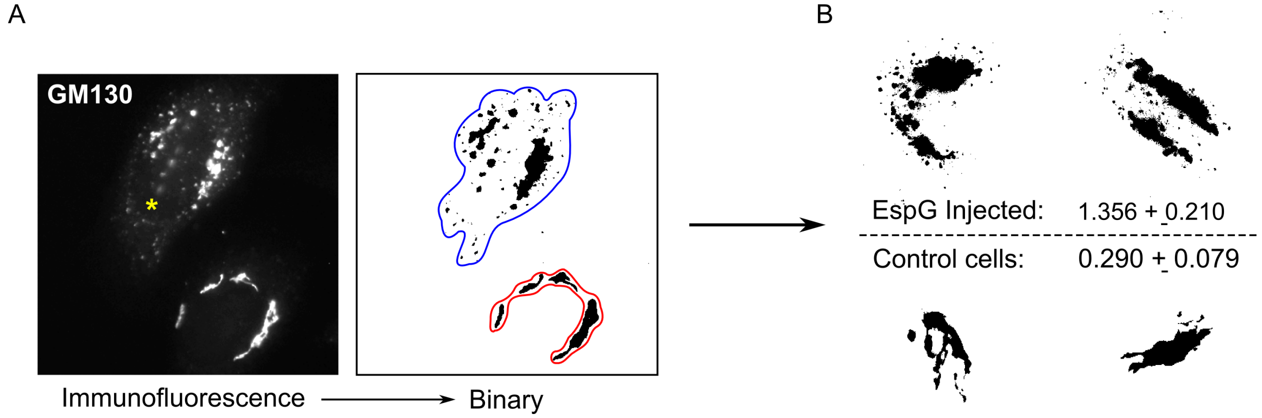

Figure S2. Mathematical definition of fragmented Golgi as applied to microinjection of EspG, related to Figure 3.

(A) Fluorescent micrographs showing Golgi marker signal (GM130). Images were converted into binary mode to obtain clear definition of positive signal. The outermost edges of Golgi marker signals were then outlined and the area measured using imaging software (ImageJ) to determine the relative spread of Golgi membranes. Area within the outlined border was considered the spread of fragmented membranes and measured in arbitrary units.

(B) Representative images of Golgi marker signal in EspG microinjected (top) or control (bottom) cells. The mean area within the border as defined in (A) is shown for microinjected and uninjected cells (n=10 cells). See supplemental Experimental Procedures explaining Golgi fragmentation analysis.

Figure S3

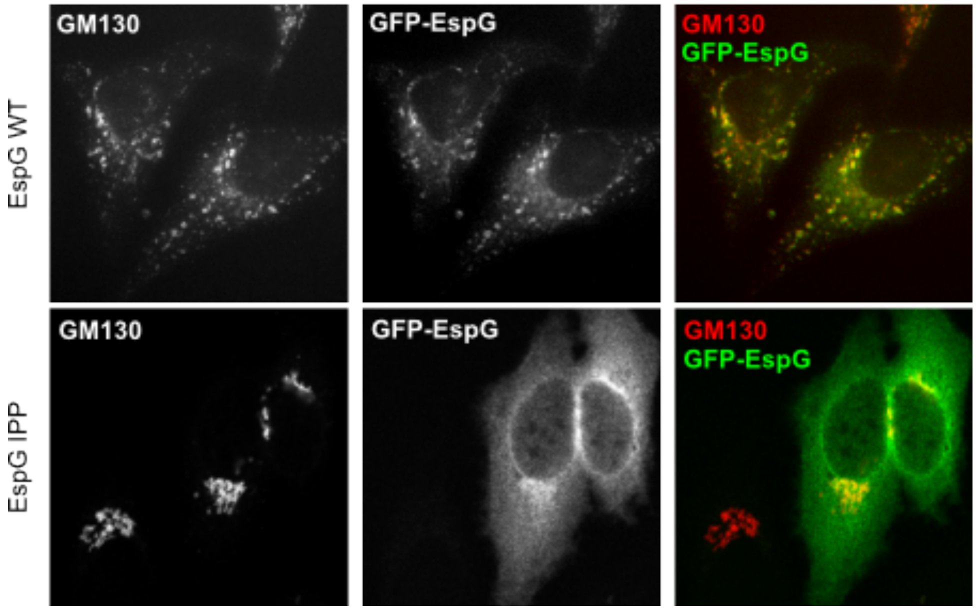

Figure S3. ARF1 binding is required for Golgi disassembly by EspG, related to Figure 3. Fluorescent micrographs showing Golgi morphology after overnight transfection with either WT or ARF1-binding deficient EspG (IPP).

Figure S4

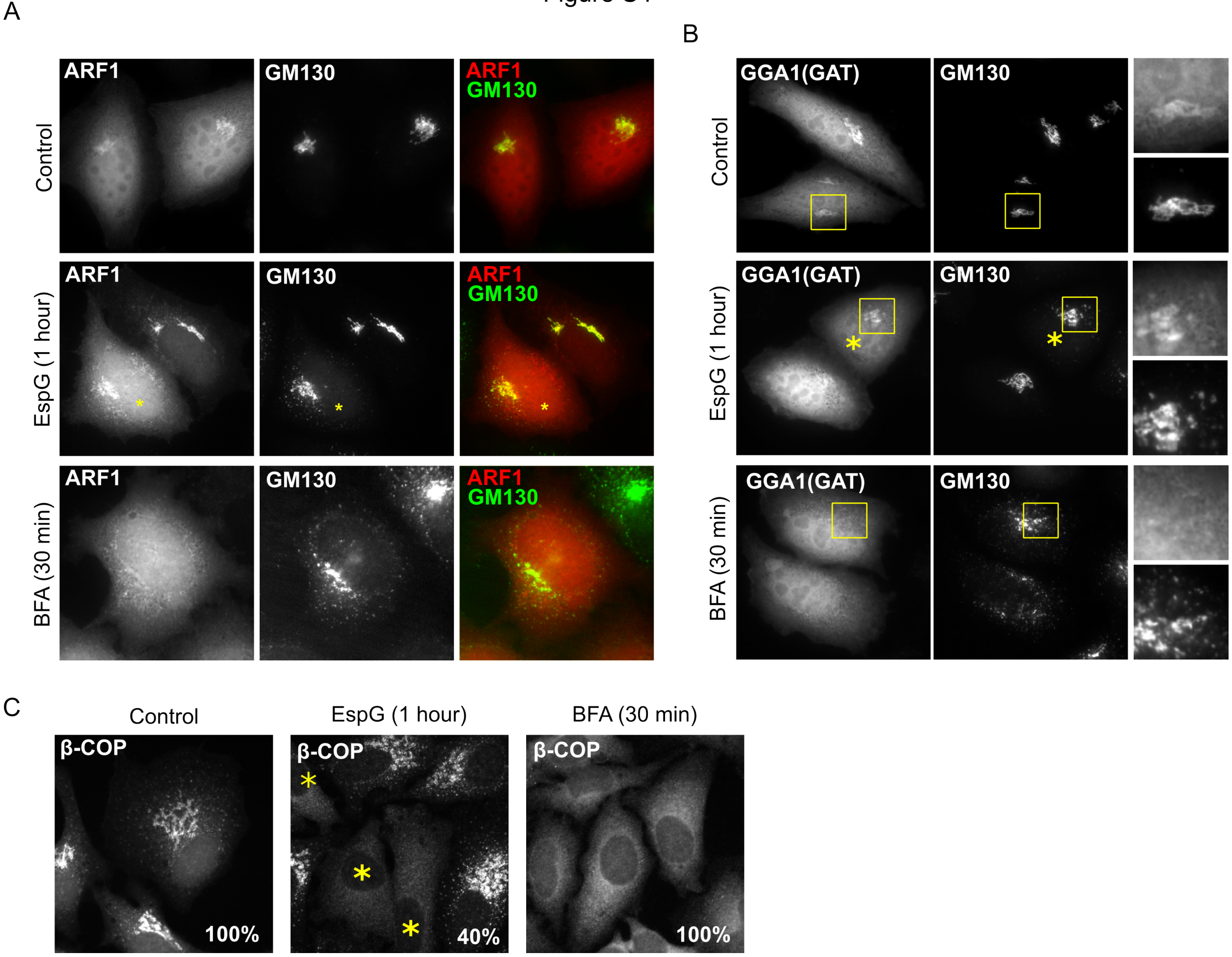

Figure S4. ARF1 remains membrane bound and active during Golgi fragmentation by EspG, related to Figure 5.

(A) ARF1-mCherry is found on fragmented membranes in cells where fragmentation is induced by EspG (asterisks), but not by BFA treatment.

(B) ARF1 is capable of recruiting downstream targets (GGA1-GAT) to membranes during Golgi disassembly by EspG. Localization of GGA1-GAT relative to Golgi membranes (GM130) is shown. Microinjected cells are marked with an asterisk.

(C) b-COP localization relative to Golgi membranes in normal cells, or after Golgi disassembly by EspG or BFA. Microinjected cells are marked with an asterisk.

Figure S5

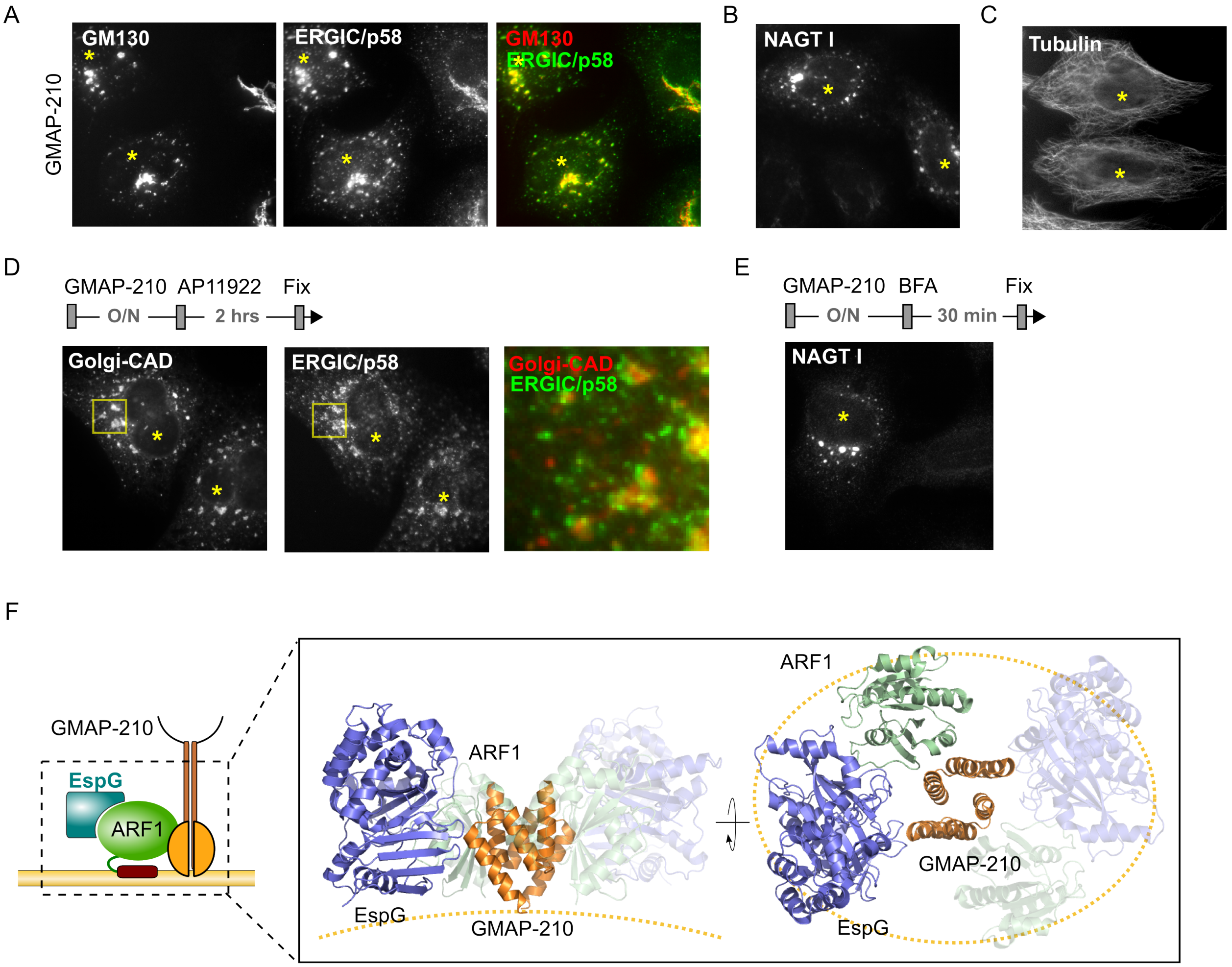

Figure S5. Overexpression of GMAP-210 closely resembles EspG function, related to Figure 6.

(A) Overexpression of GMAP-210 induces fragmentation of Golgi ribbon and enlargement of p58 clusters, similar to EspG.

(B, C) Overexpression of GMAP-210 induces accumulation of Golgi enzymes in p58 clusters and does not induce depolymerization of microtubules.

(D) Golgi-CAD escapes ER and is arrested near p58 membranes during overexpression of GMAP-210.

(E) Overexpression of GMAP-210 protects Golgi enzymes from redistribution into ER during BFA treatment.

(F) Structural model of EspG interaction with ARF1/GMAP-210 membrane tethering complex. Published structure of GMAP-210 related Golgin-245 GRIP domain bound to ARL1 (1UPT) was used to model EspG into the complex. EspG is not predicted to clash with ARF1/GMAP-210 assembly.

AUTHOR CONTRIBUTIONS

N.M.A. and A.S.S. conceived the general ideas for this manuscript. N.M.A., A.S.S. and L.E.R. planned, performed, and interpreted experiments. B.A.W. developed Golgi-CAD assay. N.M.A. and A.S.S. wrote the manuscript and all authors provided editorial input.

EXPERIMENTAL PROCEDURES

Quantification of Golgi fragmentation

A mathematical definition has been applied to accurately quantify the Golgi disassembly in microscopy assays. After converting fluorescent micrographs into binary images, the spread of GM130 marker through the cell was measured using the area contained within the outermost edges of clear GM130 signal (**Figure S2**). In fragmentation assays, Golgi was considered fragmented if the spread exceeded three (3) standard deviations from the spread measured in uninjected cells of the same experiment. Quantification data are presented in the figures.

Ligand Inducible ER-to-Golgi Trafficking Assay

The signal sequence (residues 1-81) of human β-1,4-galactosyltransferase was fused to mCherry sequence in a modified pcDNA 3.1 plasmid, followed by a furin cleavage site (FCS) and 4x repeats of FKBP F36M CAD domain (see **Figure 1A**). Cells were transfected with 0.5 μg of plasmid for 16-18 hours. Aggregation of Golgi-CAD in ER was reversed by the addition of AP21998 (2 μM final) to the media, which induced Golgi-CAD trafficking through the general secretory pathway. Final localization of Golgi-CAD was assessed 2 hours later. BFA and nocodazole treatments, as well as EspG microinjection, were performed after transfection and prior to the addition of AP21998, where indicated.

Liposome pull-downs and Rab1 GAP assay

N-terminal His-tagged ARF1 was nucleotide exchanged to GTP and incubated with Golgi mimetic liposomes containing 20 mol% DOGS-NTA (Avanti Polar Lipids). Liposomes were separated from bulk ARF1-GTP via centrifugation and resuspended in liposome binding buffer (20 mM Tris-HCl, pH 7.6, 50 mM NaCl, 10 mM $MgCl_2$). These ARF1-GTP decorated liposomes were then incubated with a combination of EspG, GGA, and ARF1GAP. To test for EspG protection of ARF1 from ARF1GAP, EspG was added 5 min prior to the addition of rat ARF1GAP. Following incubation on ice for 30 min, liposomes were sedimented and samples of the supernatant and pellet were separated on 12.5% SDS-PAGE. For Rab1 GAP assays, GST-Rab1 (1-177) (5 μM) was loaded with radio labeled $GTP\gamma P^{32}$ in loading buffer (20 mM Tris pH 7.5, 100 mM NaCl, 2 mM EDTA, 0.2 mM DTT) for 15 min at room temperature, and then

supplemented with 10 mM $MgCl_2$. It was then incubated alone, or with addition of WT or mutant EspG (1 μM) for 15 min at 30°C. Reactions have been stopped with ice cold buffer and then bound to nitrocellulose membranes. Hydrolysis of GTP was assessed by scintillation counting.
